# An Online Physical Activity Intervention for Youth With Physical Disabilities: A Pilot Study

**DOI:** 10.3389/fresc.2021.651688

**Published:** 2021-04-30

**Authors:** Ritu Sharma, Amy E. Latimer-Cheung, John Cairney, Kelly P. Arbour-Nicitopoulos

**Affiliations:** ^1^Faculty of Kinesiology and Physical Education, University of Toronto, Toronto, ON, Canada; ^2^Mental Health and Physical Activity Research Centre, University of Toronto, Toronto, ON, Canada; ^3^School of Kinesiology and Health Studies, Queen's University, Kingston, ON, Canada; ^4^School of Human Movement and Nutrition Sciences, The University of Queensland, St Lucia, QLD, Australia

**Keywords:** youth, physical disabilities, physical activity, online intervention, intervention—behavioral, social cognitive theory

## Abstract

**Background:** Physical activity (PA) interventions are limited in number and reach for youth with physical disabilities (YPD) who experience systemic barriers that may preclude their in-person participation. Further, a lack of theory in the development and evaluation of PA interventions impedes our understanding and replication of active components of behavior change. These limitations pose challenges in the effective promotion of PA in YPD. Theory-based and more inclusive methods of PA intervention delivery must be explored in our efforts to promote PA and overall health in YPD.

**Methods:** A pilot study was conducted to evaluate the feasibility and outcomes of an online, 4-week social cognitive theory-based PA intervention for YPD. Intervention feasibility (implementation fidelity, intervention compliance, and intervention acceptability) was evaluated through manual documentation, weekly feedback questionnaires, and open-ended feedback at 1-month post-intervention. Targeted social cognitive (outcome expectations, self-efficacy [task, self-regulatory, barrier] and self-regulation) and PA behavior outcomes were self-reported at baseline and 1-week and 1-month post-intervention.

**Results:** Sixteen YPD (M_age_ = 17.4 ± 2.7 years, 69% female) completed the study. Intervention feasibility was supported by high implementation fidelity (100%), high intervention compliance (>90%), and positive ratings on indicators of acceptability for all weeks of the intervention (weekly feedback questionnaire means ranging from 5.74 to 6.19 out of 7). Through open-ended feedback, participants indicated the intervention was easy to use and understand, favorably shifted their self-awareness and personal meaning of PA, and provided value and potential for future use pertaining to the learned self-regulation skills and strategies. Participants also provided formatting and content recommendations for intervention improvement. Repeated measures ANOVAs showed significant and large effect sizes for changes in participants' task (*p* = 0.01, *n*^2^*p* = 0.28) and barrier (*p* = 0.02, *n*^2^*p* = *0*.24) self-efficacy, goal-setting and planning and scheduling behaviors (*ps* < 0.001, *n*^2^*ps* = 0.42), and self-reported PA behavior (*p* = 0.02, *n*^2^*p* = 0.26).

**Conclusions:** An online PA intervention for YPD is feasible and may offer potential benefit through the enhancement of self-efficacy, self-regulation, and PA behavior. Continued research is necessary to understand the efficacy and longer-term outcomes of online, theory-based interventions for YPD as a PA promotion strategy.

## Introduction

Nearly 4% of Canadian children and youth under the age of 15 years and 13% of Canadian youth and young adults ages 15–24 years have a disability causing daily activity limitations (1, 2). Specifically, youth with physical disabilities (YPD) are at increased risk of experiencing health disparities and developing secondary health conditions related to the presence of health risk behaviors, such as insufficient physical activity (PA) (3). Despite the physical and psychosocial benefits that YPD can experience through participating in PA (4), YPD are reported to be less physically active than their typically developing peers (5) largely due to the presence of barriers to participation (6). Exploring strategies to promote PA during adolescence and young adulthood is critical, as the early adoption of positive health behaviors facilitates the maintenance of those behaviors into and throughout adulthood (7).

Existing PA interventions for YPD are limited in number and in their use of theory (8). This poses challenges for the effective promotion of PA and reinforces the need for theory-based interventions for YPD. Utilizing theory in the development and evaluation of behavior change interventions is critical for identifying and replicating the active components of an intervention leading to potential behavior change, with one of the most prominent theories in PA promotion research being social cognitive theory (SCT). Key SCT constructs that aid in our understanding and promotion of PA behavior include outcome expectations, self-efficacy, and self-regulation (e.g., goal-setting, planning, and self-monitoring) (9). According to SCT, self-efficacy is a direct determinant of behavior and has indirect effects on behavior through its influence on expectations of positive outcomes and the increased use of self-regulation strategies that are essential to achieve and maintain behavior change (9). Although limited, there is evidence supporting the use of SCT in PA interventions for youth with visual impairments (10) and cerebral palsy (11), such that youth demonstrated an increase in at least one of the targeted SCT constructs (i.e., outcome expectations, self-efficacy, and self-regulation) and in their PA behavior, respectively. Despite the absence of maintenance effects in both studies, this early evidence of short-term social cognitive and behavioral change in youth with visual impairments and cerebral palsy demonstrates that SCT may be an appropriate theoretical framework to inform the development of a PA intervention for YPD and warrants its continued exploration.

Despite the importance of theory in the development of behavior change interventions, the presence of environmental barriers to participation (e.g., inaccessible facilities, lack of available transportation) (6) also necessitates consideration of the mode of intervention delivery. The Internet may serve as an appropriate delivery alternative by eliminating environmental barriers that may preclude the participation of YPD in face-to-face PA interventions. Although online interventions have well-documented challenges related to attrition (12), their accessibility, increasingly sophisticated capabilities, and potential to engage YPD warrants further attention. Given the novelty of online theory-based PA interventions for YPD, a pilot study is necessary to understand whether future large-scale implementation could be practical and elicit meaningful change (13). Thus, this pilot study aimed to explore: (1) the feasibility of an online SCT-based PA intervention for YPD targeting outcome expectations, self-efficacy, and self-regulation; and (2) short- and longer-term changes in the targeted social cognitive and PA behavior outcomes as an initial assessment of potential intervention impact (13). Given the pilot nature of this study, hypotheses were not tested (13).

## Materials and Methods

### Intervention Design and Development

A 4-week intervention (“*Plan to Move*”*)* was developed by the first author and delivered on a web-hosting service called Weebly^©^. The structure and content of *Plan to Move* was guided in part by an existing, evidence- and SCT-based PA program for inactive adults with spinal cord injury (14). This existing program was chosen as a guide given its focus on similar SCT constructs (e.g., self-efficacy, self-regulation) and demonstrated efficacy in increasing leisure-time PA in a segment of the population with a physical disability (14). Thus, each week of the current intervention targeted a separate SCT construct, such that Weeks 1 through 4 targeted outcome expectations, task self-efficacy, self-regulation, and barrier self-efficacy, respectively.

Each week of *Plan to Move* consisted of an online session and corresponding independent activity. The content of the independent activities was adapted from the abovementioned guiding intervention and included age-appropriate modifications to the language and examples used. During these independent activities, for example, participants were asked to identify personally relevant benefits of engaging in PA (outcome expectations), reflect on positive PA experiences (task self-efficacy), engage in goal-setting, planning, and self-monitoring (self-regulation), and establish a coping plan (barrier self-efficacy).

Participants received access to and completed from home each week's online session and corresponding independent activity sequentially. Each online session included multiple webpages that participants clicked to progress through, with YouTube™ videos embedded to supplement in-text information. These embedded videos were in the format of whiteboard voiceover animations and were specifically developed for this intervention by the first author using an online video animation software called Raw Shorts. The videos varied in length, ranging from 1:00 to 2:50, in minutes and seconds (m:ss), and provided salient examples of the topics introduced in the online sessions (e.g., goal-setting and planning for PA). A manipulation check, in the form of a knowledge-based question, was included on the final webpage of each week's session. To reinforce content and encourage participants to apply the learned skills and strategies, the independent activities were to be completed after the online sessions. Each week of the intervention (i.e., online session and independent activity) was designed to be completed within ~20–25 min.

Prior to enrolling participants, adjustments (e.g., audio of YouTube™ videos, activity formatting) were made to the intervention based on feedback from two YPD in a pre-testing phase. Weekly intervention content, including the content of the YouTube™ videos, is detailed in Appendix A.

### Participants

Participants were recruited from an existing database of YPD who participated in past PA research and from a provincial organization that provides programs and services to YPD using electronic recruitment flyers. Inclusion criteria were: (a) self-reported having a physical disability; (b) aged 12–21 years, with the upper threshold of the age range reflecting the transition age (18–21 years) for youth with disabilities from child to adult rehabilitation and education services in Ontario, Canada (15); (c) able to read and speak in English; and (d) able to complete surveys over the telephone. Due to the nature of participation, exclusion criteria were: (a) self-reported visual, hearing, and/or cognitive impairment; and/or (b) participation in elite-level sport. As per the National Institutes of Health's (NIH) guidelines for feasibility and pilot studies (13), a power analysis was not conducted. Institutional research ethics approval was obtained prior to recruitment (Protocol #33624).

### Study Procedure

After confirming eligibility, informed parental consent and youth assent were provided via telephone for participants under the age of 18. Youth over the age of 18 provided informed consent. Consent and assent were documented by the researcher. Next, participants completed a demographics questionnaire and baseline assessment of the targeted social cognitive and PA behavior outcomes via telephone with the researcher delivering the intervention. The next day, participants received the link to the first online session and the corresponding independent activity in Word document format. After completing the first online session, indicated by the completion of the embedded manipulation check, participants were required to complete and return the independent activity to the researcher to gain access to the link and independent activity for Week 2. The same access-restricted procedure was followed for Weeks 3 and 4. The researcher sent text message reminders to participants if there was no indication of engagement with the online session or independent activity 5 days after receiving intervention materials or once they reached the threshold for “late” completion of the respective week of the intervention. Other than the researcher's involvement in the delivery of intervention materials, delivery of text message reminders, and availability to troubleshoot technical issues or answer questions, participants' engagement in the intervention was entirely self-led. Participants were scheduled to complete the same assessment that was administered at baseline with the same researcher via telephone 1 week and 1 month after completing the Week 4 online session and independent activity. The administration of the baseline and post-intervention assessments took ~30–45 min to complete.

### Measures

#### Demographics

Participants reported their age, gender, ethnicity, height, weight, disability type and duration, and use of a mobility device.

#### Intervention Feasibility

Aligning with the NIH Framework for Developing and Testing Mind and Body Interventions (13), feasibility was conceptualized as: (a) *implementation fidelity (dose and adherence)*, (b) *intervention compliance*, and (c) *intervention acceptability*. *Implementation fidelity* (i.e., delivered *dose*) and *intervention compliance* (i.e., online session and independent activity completion) were dichotomized as “complete” or “incomplete” for each week. “Complete” indicated that the session was delivered by the researcher to the participant (*dose*) and the manipulation check and independent activity were completed by the participant (*intervention compliance*). *Adherence* to the delivery schedule (i.e., each week was delivered within 7 days of participants completing the previous week) and participants' timely completion of the online sessions and independent activities (i.e., within 7 days of receiving access) were dichotomized as “on-time” or “late”. Mean view time duration in minutes and seconds (m:ss) and mean view time percentage of each YouTube™ video was extracted from YouTube™ Analytics. *Intervention acceptability* was evaluated through feedback questionnaires completed by participants at the end of each week's independent activity. Participants rated on a Likert scale of 1 (strongly disagree) to 7 (strongly agree) whether each week of the intervention was: interesting, easy to understand, taught them new and trustworthy information, easy to navigate, and presented information and strategies that were helpful (16). Weekly acceptability scores were calculated as means of participants' ratings of these parameters. For additional detail on intervention acceptability, participants provided open-ended feedback during the 1-month post-intervention assessment on program satisfaction, utility, potential impact, and recommended improvements.

#### Social Cognitive and Behavioral Outcomes

##### Outcome Expectations

Participants completed an 11-item adjectival instrument on their personal beliefs about particular outcomes occurring as a result of engaging in PA and the value they place on those particular outcomes (e.g., “Physical activity will help me to have an adventure,” and “Being adventurous is fun.”) (17). Items were rated on a scale of 1 (never) to 6 (always). This scale has demonstrated acceptable reliability among typically developing youth (17). To reduce participant burden, the measure was truncated from 23 to 11 item pairs to only include outcome expectations that were targeted in the intervention.

##### Task Self-Efficacy

Participants completed a 7-item instrument on their context-specific confidence to engage in PA (e.g., “I can be physically active during my free time on most days.”). Items were rated on a scale of 1 (disagree a lot) to 5 (agree a lot). This instrument has demonstrated acceptable test-retest reliability and factorial validity in typically developing youth (18). While this measure has been used to assess task self-efficacy for PA in youth with CP (11), psychometric properties were not specified.

##### Self-Regulatory Efficacy

Two components of self-regulatory efficacy were evaluated: goal-setting self-efficacy and planning and scheduling self-efficacy. To examine goal-setting self-efficacy, participants completed a 4-item instrument on their confidence in their ability to set PA goals in the next 4 weeks (e.g., “How confident are you that you can set realistic goals for maintaining your physical activity for the next 4 weeks?”) (21). To examine planning and scheduling self-efficacy, participants completed a 7-item instrument on their confidence in their ability to schedule a self-managed PA routine in the next 4 weeks (e.g., “How confident are you that you can arrange your schedule to do physical activity each week no matter what for the next 4 weeks?”) (21). Items from both instruments were rated from 0 (not at all confident) to 100 (completely confident). The language of both instruments was modified, such that “independent physical activity” was replaced with “physical activity.” Both instruments have demonstrated acceptable reliability and validity in adults with spinal cord injury (20).

##### Barrier Self-Efficacy

Participants completed an 8-item scale on their confidence to overcome barriers that may prevent them from engaging in PA (e.g., “Assuming you are very motivated, how confident are you that you could participate in physical activity if you feel tired?”). Items were rated on a scale of 1 (not confident at all) to 7 (completely confident). Six of these eight items have demonstrated high internal consistency in typically developing children and youth (19). Two additional items relating to transportation problems and a lack of support were included, as these are salient PA barriers that individuals with physical disabilities often encounter (6). These items have demonstrated acceptable internal consistency in adults with spinal cord injury (20).

##### Self-Regulation Behavior

Two self-regulation behaviors were evaluated: goal-setting, and planning and scheduling. Participants completed the 10-item Exercise Goal Setting Scale (EGS) (22) and the 10-item Exercise Planning and Scheduling Scale (EPS) (22). Items from both the EGS and EPS were rated on a scale of 1 (does not describe) to 5 (describes completely). Examples from the EGS and EPS include, respectively: “I have developed a series of steps for achieving my physical activity goals,” and “Physical activity is generally not a high priority when I plan my schedule.” The EGS and EPS have demonstrated good internal reliability among college-aged youth (22). The language of the EGS and EPS was modified by replacing “exercise” with “physical activity.”

##### Physical Activity

Participants completed the 6-item Leisure-Time Physical Activity Questionnaire for People with Spinal Cord Injury (LTPAQ-SCI), where they were asked to self-report the number of days and minutes on those days spent engaging in mild-, moderate-, and heavy-intensity PA, during their leisure time in the past 7 days (23). Weekly minutes of mild-, moderate-, and heavy-intensity PA were summed for a total amount of weekly minutes of PA overall. The LTPAQ-SCI has been found to be valid and reliable for persons with physical disabilities (23, 24).

### Statistical Analysis

Statistical analyses for quantitative data were performed using SPSS Version 24.0. Descriptive statistics were conducted to summarize participants' demographic characteristics and quantitative feasibility outcomes measured through manual documentation and weekly feedback questionnaires. One-way repeated measures analyses of variance (RM-ANOVAs) were performed to assess social cognitive and PA behavior change from baseline to 1-week and 1-month post-intervention. Model assumptions were tested and the Greenhouse-Geisser correction was used for violations of the within-subjects assumption of homoscedasticity. Bonferroni corrections were performed to determine significant change(s) between the three time points. Given the absence of a power analysis, effect sizes were also used in the interpretation of results, such that η^2^*p*s of 0.01, 0.06, and 0.14 represented small, medium, and large effect sizes, respectively (25).

Open-ended feedback from the 1-month post-intervention assessment was transcribed verbatim, de-identified through the assignment of pseudonyms and removal of identifying information, and underwent content analysis (26) by the first author. Transcripts were coded inductively to establish categories within the four topics, which were agreed upon by a critical friend (KPAN). Disagreements in the labeling of emerging categories or coding were resolved through discussion leading to consensus. The frequency of key words and phrases pertaining to each category was recorded.

## Results

### Participant Flow and Characteristics

Of the 33 eligible youth, two declined to participate (6%), 11 (33%) did not respond to the follow-up email, and 20 (61%) enrolled in the study. Sixteen youth (80%) completed the study in its entirety and four (20%) were lost to follow-up (data excluded from analyses). These four participants did not respond to emails regarding their continued participation in the study. Table 1 provides participants' demographic characteristics.

**Table 1:** Participant characteristics (*N* = 16).

**Characteristic**	**Value**
Age (years)	
M (SD)	17.4 (2.7)
Range	13–21
Sex, *n*	
Male	5
Female	11
Body Mass Index (kg/m^2^), M (SD)	21.92 (6.55)
Ethnicity, *n*	
White	11
East Asian	2
Other (Black, South Asian, West Asian)	3
Type of Physical Disability, *n*	
Cerebral palsy	3
Muscular dystrophy	3
Neuromuscular disorder	2
Spinal cord injury	4
Other (brain injury, stroke, genetic disorder)	4
Years Living with Physical Disability, M (SD)	11.2 (6.7)
Use a Mobility Device, *n*	14
Manual wheelchair	4
Power wheelchair	5
Cane	2
Crutches	1
Other	2

*M, mean; SD, standard deviation*.

### Intervention Feasibility

Table 2 summarizes implementation fidelity and intervention compliance outcomes.

**Table 2:** Implementation fidelity and intervention compliance outcomes.

**Intervention component**	**Implementation fidelity**			**Intervention compliance**			
	**Dose, *n***	**Number of days to deliver,^a^ M (SD) [Range]**	**Adherence: On-time delivery, *n***	**Online session completion, *n***	**Independent activity completion, *n***	**Number of days to complete,^b^ M (SD) [Range]**	**On-time completion of each week, *n***
Baseline^c^	16	–	16	–	–	–	–
Overall^d^	100%	4.35 (0.41)	98%	94%	98%	5.28 (4.02)	81%
Week 1	16	–	16	16	16	3.18 (2.32) [0–7]	16
Week 2	16	4.00 (1.93) [0–8]	15	14	16	5.88 (3.59) [0–13]	12
Week 3	16	4.25 (2.08) [0–7]	16	15	15	5.38 (4.15) [0–15]	12
Week 4	16	4.81 (2.34) [0–7]	16	15	16	6.69 (5.03) [1–15]	12
1-week post-intervention^c^	16	6.94 (4.02) [3–15]	15	–	–	–	–
1-month post-intervention^c^	16	22.31 (4.22) [17–30]	14	–	–	–	–

*M, mean; SD, standard deviation*.

a *Number of days between participants' completion of each week of the intervention and delivery of the following week*.

b *Number of days for participants to complete each week upon receiving materials (i.e., link to online session and independent activity) via email*.

c *Refers to the three assessments conducted by the researcher and do not represent weekly sessions*.

d *For outcomes referring to number of days, values reflect an overall average of the mean number of days to deliver and complete each week of the intervention. Percentage values reflect the overall percentage of timely delivery, online session and independent activity completion, and timely completion of each week of Plan to Move out of the 64 total instances of intervention delivery (4 weeks × 16 participants)*.

#### Implementation Fidelity

The intervention was delivered in its full *dose* (100%), meaning all online sessions and independent activities were delivered to all participants. Concerning *adherence*, all 4 weeks were delivered within an average of 4.35 (SD = 0.41) days after completion of the previous week's online session and independent activity. There was one instance of late delivery due to technical difficulties. In addition, the baseline and 1-week and 1-month post-intervention assessments were administered to all participants (100%). Adherence to the delivery schedule of the 1-week and 1-month post-intervention assessments was achieved, but was constrained by scheduling challenges in 3 of 16 participants. Overall, the intervention and assessments were delivered as intended.

#### Intervention Compliance

Overall, 13 of 16 participants completed all of the online sessions, indicated by the completion of the embedded manipulation check. In addition, 15 of 16 participants completed all of the independent activities. Out of the 64 total instances of intervention delivery to all participants (4 weeks x 16 participants), the overall percentage of completion of the online sessions and independent activities was 94% and 98%, respectively. Participants took, on average, 5.28 (SD = 4.02) days to complete each week of the intervention upon receiving access. Overall, 81% of the total delivered weeks of the intervention were completed on-time. Instances of “late” completion occurred between Weeks 2 and 4, with four participants not completing the respective online session and/or independent activity within 7 days of receiving access. On instances of “late” completion, participants took, on average, 4.33 (SD = 2.58) additional days to complete the online session and/or independent activity.

Participants viewed, on average, 69% of the total minutes (7:33 of 11:00) of the YouTube™ videos. The average view time percentages of each of the seven videos ranged from 61% to 86%. The “Welcome” video in Week 1 was viewed for the longest duration (86% [1:02 of 1:12]), whereas the “Goal-Setting” and “Scheduling” videos in Week 3 had the lowest view time percentages (61% [1:44 of 2:50] and 67% [1:01 of 1:31], respectively).

#### Intervention Acceptability

##### Weekly Feedback Questionnaires

**Figure 1:**
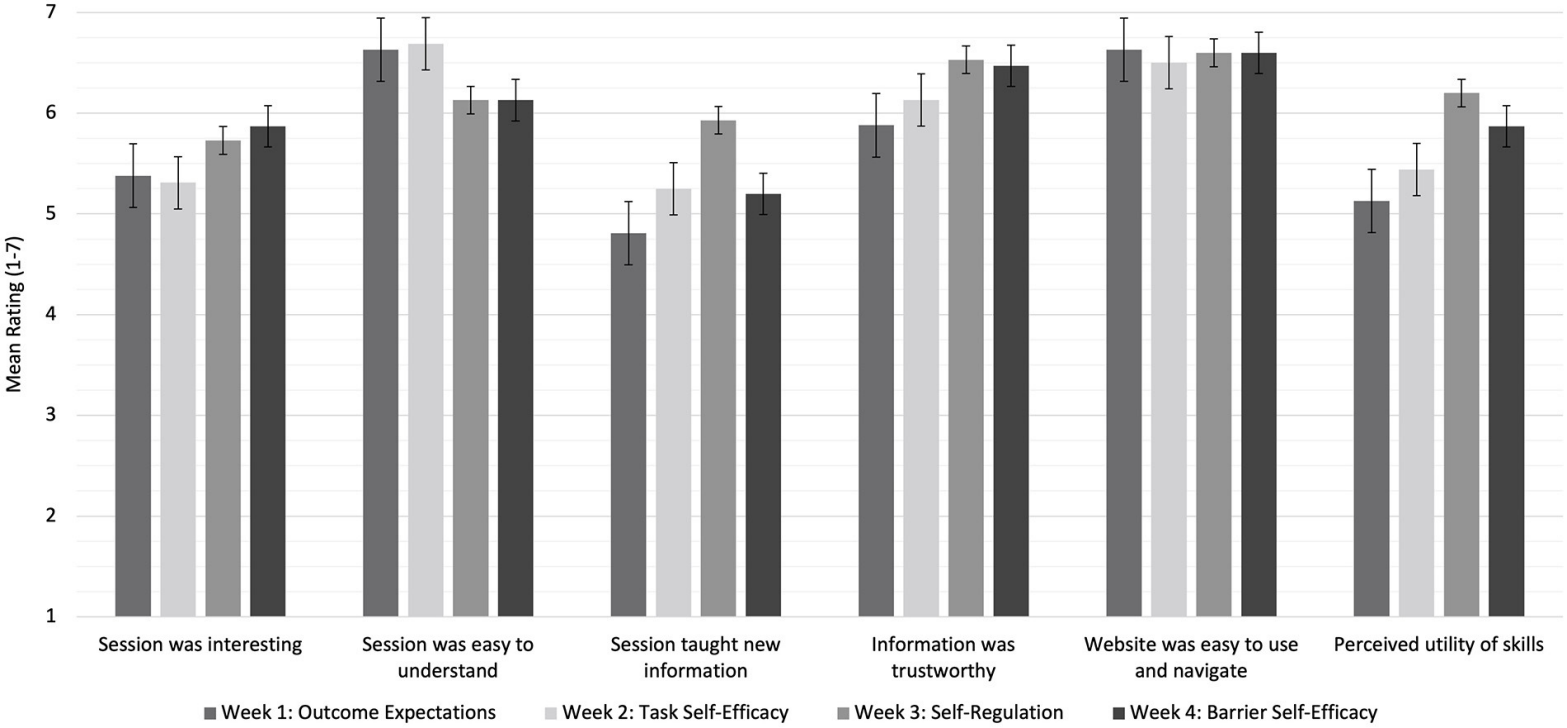
Mean weekly ratings of intervention acceptability parameters.

For each week of the intervention, participants provided positive ratings (i.e., scores above the “neutral” anchor point) on all parameters of acceptability (ratings ranging from 4.81 to 6.69 out of 7). Figure 1 presents mean ratings of each parameter of acceptability for each week of the intervention. The overall mean acceptability for Weeks 1 through 4 was 5.74 (SD = 0.77), 5.89 (SD = 0.64), 6.19 (SD = 0.34), and 6.02 (SD = 0.50), respectively.

##### Open-Ended Feedback

Categories, frequencies, and quotes emerging from the content analysis are presented in Table 3. Overall, participants indicated that *Plan to Move*: (1) was easy to use and understand; (2) favorably shifted their self-awareness and personal meaning of PA; and (3) provided value and potential for future use pertaining to the learned skills and strategies. Participants' recommendations for improvements related to formatting of the independent activities, including more examples on self-regulation, and providing information about PA guidelines, sample exercises, and accessible facilities and sport opportunities.

**Table 3:** Open-ended feedback content analysis summary.

**Topic**	**Category**	**Frequency (% of total)**	***n***	**Sample quote**
Satisfaction	User-friendliness	35 (18%)	16	“I really liked the websites. The websites were working. […] I liked how this one was working good, I just clicked a link and got there. It was easy to use.”—Sophie
				“I thought they were really easy to use because there weren't a lot of different links, and only one button I had to press when I got through one section.”—Leah
	Clarity	38 (19%)	16	“One thing I noticed that really nice was the videos. I liked how they summed everything up really nicely. Like from reading the website to watching the video, it made it more understandable.”—Logan
				“[…] there wasn't a lot of information on one page, so it wasn't overwhelming. Each section had the right amount of information. The activities, like the way they were explained, it was broken down really well, and I understood exactly what I had to do. It helped me understand the information you have on the website and put it into context for me.”—Leah
Potential impact	Increased self-awareness of their PA	25 (13%)	12	“When you're talking about incorporating physical activity into a busy schedule [*in Week 3*], it really helped a lot because I do have a busy schedule. Now I can find key points where I can fit physical activity here or there or whenever. It changed how I looked at things and how much time I have.”—Grace
				“When there's a day when I realize maybe I'm doing too much, I can schedule physical activity for other days when I'm not doing as much. Especially with the chart [a self-monitoring tool provided in Week 3], it was really easy to see where I could plan my physical activity.”—Molly
	Positive reframing of PA	13 (7%)	7	“I think what I realized the most was that physical activity is not just going to the gym or playing a certain sport. Like there's a lot of things you can do in your daily life that can count for physical activity, like into your daily routine without having to disrupt it. Like taking longer walks. After this program, I took up one new sports activity. I got inspired to learn [something new], so I started taking ice skating classes. It's really fun—it's challenging but I'm just going at a slow pace and I'm having fun.”—Amanda
				“By only doing 10 min at a time, it makes it more manageable and less intimidating.”—Charlotte
Utility	Value of learned skills	24 (12%)	11	“A big thing for me is not having time. These skills help me understand how to plan to have more time to be active. Also, this program reinforced the idea of regular goal-setting and how it can help me get more physical activity.”—Ethan
				“Scheduling helped me see when I had free time. I knew I could use that time to do physical activity. It made sure I wasn't sitting around and wasting time.”—Zara
	Current and future use of learned skills and strategies	30 (15%)	14	“I think I'd use the things I learned to continue working out. Like the reminders definitely are a huge help and have been something I've been using since I learned about them. Instead of just putting it in my calendar and forgetting to do it, putting it in my calendar and setting reminders really helped me remember to actually go and achieve that goal or workout that I wanted to do.”—Camila
				“I liked having a set plan and sticking to that plan. I put reminders on my phone to stretch during homework breaks, or while watching TV. I liked it, because I need to do those stretches for my spasms anyway.”—Chloe
Recommended improvements	Formatting	15 (8%)	13	“I found the first few were properly formatted, […] but there were a few pictures covering the questions. Maybe have like on the website that you used […], have it on the website so then you don't have the document where the formatting gets messed up.”—Logan
				“I was having some issues with formatting. I would not recommend doing the activities on Word. If there was like an online program that would work universally, that would probably be better.”—Olivia
	Additional information	15 (8%)	7	“I would like to know more about nutrition and stretching and all that kind of stuff after physical activity, like how to take care of your body if you're sore. How often you should be exercising, as in like when you should take a rest, and rotating muscle groups, would have been really helpful. […] This program may be good for teenagers or like younger teens who haven't yet been educated on the benefits of physical activity. I think for me, it may be really nice if the program had links to sport associations to get you involved or accessible facilities to stay fit. Sample exercises, that kind of stuff.”—Olivia
				“You could give us links to gyms where we could go or something like that. Maybe accessible places where we can go workout. It's not always helpful to just tell us about the tools to get active, but you have to kind of reinforce that with where we can get active.”—Elliot
				“I think like when you're talking about goal-setting, there were some really good examples, but I just feel like maybe if you were like a little more descriptive it would be better.”—Grace
				“[…] providing more scientific information for some things in Week 1 [*referring to outcome expectations]*, like some studies or background. You see the value for those things. That would also help with motivation and drive to start being active.”—Logan

*All participants were assigned pseudonyms to protect their anonymity*.

### Social Cognitive and Behavioral Outcomes

Table 4 summarizes the means, standard deviations, and RM-ANOVAs for all social cognitive and PA behavior outcomes. There were significant and large effect sizes showing increased task (*n*^2^*p* = 0.28) and barrier self-efficacy (*n*^2^*p* = 0.24) between baseline and 1-month post-intervention (adjusted *p*s = 0.01 and 0.04, respectively). There were significant and large effect sizes showing increased goal-setting and planning and scheduling behaviors (*n*^2^*p*s = *0*.42) between baseline and 1-week post-intervention (adjusted *p*s = 0.01), and from baseline to 1-month post-intervention (adjusted *p*s = 0.01). Despite a significant main effect, significant *post-hoc* effects were not found for goal-setting self-efficacy (all adjusted *p*s > 0.05). No significant changes were found in outcome expectations or planning and scheduling self-efficacy. Lastly, there was a significant and large effect size showing increased self-reported PA behavior (*n*^2^*p* = 0.26) between baseline and 1-month post-intervention (adjusted *p* = 0.04).

**Table 4:** Means, standard deviations, and one-way RM-ANOVAs for social cognitive outcomes and self-reported PA.

**Variable [Potential score range]^a^**	**Baseline, M (SD) [Actual range]**	**1-week post, M (SD) [Actual range]**	**1-month post, M (SD) [Actual range]**	***F*-ratio (df)**	***p***	**η^**2**^*p***
1. Outcome expectations [22–396]	264.69 (61.44) [164–396]	290.56 (65.75) [168–396]	281.50 (61.08) [163–396]	2.66 (2, 11)	0.09	0.15
2. Task self-efficacy [1–7]	3.43 (0.61) [2.29–4.57]	3.79 (0.45) [2.86–4.29]	3.81 (0.59) [2.57–4.57]	5.89 (2, 11)	0.01	0.28
3. Goal-setting self-efficacy [0–100%]	69.84 (14.56) [45.00–95.00]	77.47 (15.85) [51.25–100.00]	75.70 (11.39) [57.50–95.00]	4.22 (2, 11)	0.02	0.22
4. Planning and scheduling self-efficacy [0–100%]	72.19 (17.05) [32.14–96.43]	75.96 (16.54) [38.57–98.57]	75.89 (14.13) [40.00–92.14]	1.79 (2, 11)	0.20	0.11
5. Barrier self-efficacy [1–7]	4.29 (0.90) [2.75–5.88]	4.83 (1.14) [2.63–6.50]	4.81 (1.08) [3.38–6.88]	4.66 (2, 11)	0.02	0.24
6. Goal-setting behavior [10–50]	28.69 (7.27) [17–39]	34.44 (8.49) [18–46]	35.31 (6.06) [19–44]	11.01 (2, 11)	<0.001	0.42
7. Planning and scheduling behavior [10–50]	28.44 (8.64) [16–45]	34.38 (8.02) [19–49]	33.88 (6.49) [20–45]	10.66 (2, 11)	<0.001	0.42
8. Self-reported weekly minutes of PA	248.13 (171.34) [50–590]	320.75 (184.64) [40–670]	415.94 (365.46) [45–1,185]	5.32 (2, 11)	0.02	0.26

a *higher scores reflect improved outcomes for each variable*.

*M, mean; SD, standard deviation*.

*η^2^p, partial eta squared, such that 0.01 = small, 0.06 = medium, and 0.14 = large effect sizes (25)*.

## Discussion

This pilot study explored the feasibility and potential social cognitive and behavioral outcomes of an online SCT-based PA intervention for YPD. Intervention feasibility was supported by: (1) high implementation fidelity, (2) high intervention compliance, (3) positive ratings on indicators of acceptability, and (4) participants' perceived satisfaction, impact, and utility of the intervention. Participants experienced significant and large-sized increases in task and barrier self-efficacy, goal-setting, planning and scheduling, and self-reported PA. These findings are encouraging and demonstrate that an SCT-based online PA intervention for YPD is feasible and may yield positive social cognitive and behavioral change over a short period.

Given their novelty, the feasibility of online PA interventions for YPD was largely unknown. This study contributes to the generation of knowledge on related feasibility outcomes and can inform the procedure of future online PA interventions for YPD. Notably, nearly perfect implementation fidelity was achieved, demonstrating that the delivery of an online PA intervention as intended in YPD is practical. The digital and self-led nature of the intervention reduced the magnitude of the facilitator's involvement substantially in comparison to the degree of involvement that could be expected in traditional in-person or facilitator-led interventions. This reduced level of facilitator involvement, and potential burden, was likely a contributing factor to the high implementation fidelity observed in the current study. As such, these findings provide further support for the value of leveraging technology in the delivery of PA interventions for YPD.

Considering the challenges related to retention in technology-based interventions (12), high intervention compliance (>90%) and relatively low attrition (20%) in the current study is promising. Text message reminders (11) and the short duration of the intervention (27) may have facilitated greater compliance and retention than a longer intervention would have. In addition, the self-led nature of the intervention may have allowed participants some degree of flexibility in comparison to a traditional in-person or facilitator-led intervention, where scheduling or other constraints may lead to poor compliance or attrition. In contrast, in the current intervention, participants were given a certain degree of autonomy to complete each week of the intervention (i.e., within 7 days). Further, given the frequent use of YouTube™ by YPD (28), embedding YouTube™ videos may have offered a salient method of communication that encouraged some degree of continued engagement. The use of YouTube™ videos was novel, and thus, expected outcomes relating to its feasibility were unknown. Despite 69% average viewership of the total minutes of the embedded videos, participants' positive response to the YouTube™ videos, as demonstrated through their open-ended feedback during the 1-month post-intervention assessment, suggests that it may be worthwhile to incorporate YouTube™ videos in future PA interventions for YPD. Further work is needed though to determine the appropriate video length and content (e.g., knowledge vs. examples demonstrating the application of skills) to optimize YPD's sustained engagement and exposure to intervention content.

With regard to intervention acceptability, participants provided positive ratings (i.e., scores above the Likert scale's “neutral” anchor point) on all indicators of acceptability in the weekly feedback questionnaires. Overall, participants indicated that each week of *Plan to Move* was easy to navigate, easy to understand, and provided credible information that they would likely use in the future to manage their PA behavior. Notably, participants' ratings of the perceived novelty and utility of learned skills was highest in Week 3, which targeted self-regulation. Similar intervention acceptability outcomes were revealed through participants' open-ended feedback provided during the 1-month post-intervention assessment. Participants shared that, overall, they were satisfied with *Plan to Move*, largely as a result of the intervention's user-friendliness (e.g., simple navigation) and clarity. Furthermore, participants indicated that the learned self-regulation skills would likely help them manage their PA behavior in the future. Overall, participants' open-ended feedback aligned with the findings from the weekly feedback questionnaires.

Despite their overall perceived acceptability of *Plan to Move*, participants also provided valuable recommendations on how to improve future content and design. In particular, older participants (aged 20–21 years) expressed interest in learning about PA guidelines, sample exercises, and resources on accessible facilities and sport opportunities. Although PA prescription was not within the scope of the intervention, these suggestions provide insight on the type of information older youth may be seeking when participating in a PA intervention. Participants also indicated a preference for the independent activities to be embedded within the online sessions rather than as an offline Word document to mitigate challenges related to formatting incompatibilities across different operating systems and versions of software. Thus, future PA interventions for YPD should streamline all components of the intervention within one interface to deliver a more integrated experience with less potential for formatting incompatibilities.

Participants' enhanced social cognitions was a positive outcome. Contrary to previous evidence (11), task self-efficacy increased. This discrepancy may be explained by the provision of various self-regulatory strategies in the current intervention. Emphasizing self-regulation may have counteracted potential negative effects on self-efficacy by providing participants with a set of tools to manage salient PA-related challenges that were potentially heightened by participating in the intervention itself. From a theoretical perspective, self-efficacy is a direct determinant of health behavior and also has indirect effects on behavior through intermediate determinants (e.g., self-regulation). Thus, future PA interventions should target self-efficacy *and* self-regulation to maximize potential for behavior change. The observed increase in barrier self-efficacy is consistent with previous evidence (10) and should continue to be targeted.

Although goal-setting and planning and scheduling behaviors increased, this was not complemented by an increased self-efficacy to engage in those behaviors. This observation warrants consideration of the role of parents of YPD. Given the unique challenges that YPD experience, parents are a vital source of support and often manage their child's schedule and act as a prompt to execute plans (29). Thus, YPD may not feel confident in their ability to self-manage goals. Shifting the responsibility of self-regulation from parent to child can enhance independence and better prepare YPD to self-manage their PA. Thus, self-regulation should be targeted in such a way that also enhances YPD's self-efficacy to engage in self-regulation behaviors.

Contrary to previous evidence (10), outcome expectations did not increase. Participants' open-ended feedback suggests that outcome expectations may need to be targeted differently in YPD. For example, participants expressed interest in learning about the scientific literature supporting the benefits of PA. This approach may substantiate the benefits of PA and be more effective than listing benefits that YPD are likely aware of. Future work should explore how outcome expectations can be more effectively targeted and enhanced in YPD, as SCT constructs are reciprocally interrelated and have direct effects on behavior (9).

Increased self-reported PA between baseline and 1-month post-intervention was an unexpected but welcomed outcome, as previous evidence demonstrated no significant increases in self-reported or objectively measured PA following intervention in YPD (11). Targeting known theoretical correlates of PA for youth in the current study may have facilitated an increase in PA. Despite the LTPAQ-SCI being a validated measure of PA in persons with physical disabilities (24), participant knowledge of the intervention's objective to enhance PA may have caused response bias and warrants caution in the interpretation of this observed increase. Although utilizing wearable devices (e.g., accelerometer) for the measurement of PA would counter such bias, this approach would pose challenges in the reliable measurement of PA in non-ambulatory YPD (30).

This was the first study to evaluate the feasibility and outcomes of an online theory-based PA intervention in a diverse sample of YPD. Focusing on the end-user in the current study allowed for an understanding of what elements of the intervention did and did not work from a usability and feasibility perspective. Further, the use of theory allowed for insight on constructs that were enhanced and others that may need to be targeted differently in YPD (i.e., outcome expectations and self-regulatory efficacy).

Despite these strengths, the lack of a control group precludes the determination of whether the observed changes would or would not have occurred in the absence of an intervention. Thus, the observed changes in participants' social cognitive and behavioral outcomes are not an indication of intervention efficacy nor can they be attributed as an outcome of the intervention itself. Furthermore, although discussion topics were introduced neutrally and participants were unaware of who developed the intervention, there was potential for bias in participants' open-ended feedback, as the discussions were conducted by the researcher delivering the intervention. Future implementation at a larger scale should be appropriately powered and include a control group and longer follow-up period to minimize sampling bias, enhance generalisability, determine efficacy, and elucidate longer-term outcomes. In consideration of implementation at a larger scale, although the use of technology in the current study mitigated the environmental barriers that may otherwise preclude YPD from their in-person participation in PA interventions, it is important to acknowledge that this intervention delivery approach can pose an alternative set of barriers related to inequitable access to technology (e.g., computer, Internet, and software licenses). Although access to technology did not pose any challenges in the current study, strategies to address these potential barriers in the emergence of progressively technology-based intervention approaches must be considered to manage social inequities and deliver a comprehensive PA promotion strategy to YPD that minimizes the impact of a spectrum of barriers and does not drive further health inequities.

Findings from this study support feasibility and can guide the development and implementation of future online PA interventions for YPD. Participants' enhanced social cognitive and behavioral outcomes demonstrates the potential benefit that YPD may experience from participating in an intervention of this nature. Continued research on the topic of online theory-based PA interventions is critical for creating high-quality opportunities for YPD to learn strategies that enable them to enhance and self-manage their PA and overall health. These benefits may not otherwise be accessible to YPD without this alternative method of intervention delivery, which should therefore be considered in the development of future PA promotion strategies for this population.

## Data Availability Statement

The raw data supporting the conclusions of this article will be made available by the authors, without undue reservation.

## Ethics Statement

The studies involving human participants were reviewed and approved by the Health Sciences Research Ethics Board at the University of Toronto. Verbal informed parental consent and verbal youth assent were provided for participants under the age of 18 to participate in this study, and participants over the age of 18 provided verbal informed consent. Verbal informed consent and assent were documented.

## Author Contributions

RS designed the study with input from AEL-C, JC, and KPA-N. RS developed and delivered the Plan to Move intervention, recruited participants, collected and analyzed data, and lead the writing of the manuscript. KPA-N supervised the execution of the study. All authors contributed to the review, editing, and final approval of the manuscript.

## Conflict of Interest

The authors declare that the research was conducted in the absence of any commercial or financial relationships that could be construed as a potential conflict of interest.

## Supplementary Material

Appendix A Content overview of weekly sessions, YouTube™ videos, and independent activities of *Plan to Move*.

## Abbreviations

PA: physical activity
LTPAQ-SCI: Leisure-Time Physical Activity Questionnaire for People with Spinal Cord Injury
RM-ANOVA: repeated measures analyses of variance
SCT: social cognitive theory
YPD: youth with physical disabilities.
