# Supplementary material for: An Online Physical Activity Intervention for Youth With Physical Disabilities: A Pilot Study
Source: Front Rehabil Sci. 2021 Apr 30;2:651688. doi: 10.3389/fresc.2021.651688 (PMC9397683; doi:10.3389/fresc.2021.651688)
Supplement: Appendix A — Content overview of weekly sessions, YouTube™ videos, and independent activities of Plan to Move. [file Table_1.DOCX]

| **Appendix A.** Content overview of weekly sessions, YouTube™ videos, and independent activities of *Plan to Move* | | | | |
| --- | --- | --- | --- | --- |
| Week | Construct | Online session | YouTube™ video topic (m:ss) | Independent activity |
| 1 | Outcome expectations | - Introducing physical, social, and self-evaluative outcome expectations of physical activity - Prompting thought about personally relevant benefits of physical activity | - Overview of *Plan to Move* (1:12) - Physical and psychosocial benefits of physical activity (1:14) | - Defining physical activity - Identifying personally relevant short- and long-term benefits of physical activity |
|  |  |  |  |  |
| 2 | Task self-efficacy | - Broadening the definition of physical activity to include shorter and more manageable bouts - Learning about the importance of setting realistic, short-term goals to maximize and enjoy regular success in a physical activity context | - Redefining physical activity to include a variety of activities (1:31) - How to segment daily physical activity into more manageable bouts (1:42) | - Sharing if and how participants’ definition of physical activity changed from last week - Sharing positive accomplishments related to physical activity to increase confidence |
|  |  |  |  |  |
| 3 | Self-regulation | - Introducing self-regulation strategies (i.e., goal-setting, scheduling, and self-monitoring) and their value in a physical activity context | - An example of setting a S.M.A.R.T. physical activity goal (2:50) - An example of scheduling time to achieve a physical activity goal (1:31) | - Engaging in self-monitoring, setting S.M.A.R.T. physical activity goal, and scheduling time to achieve physical activity goal - Identifying personal motivations for increasing physical activity |
|  |  |  |  |  |
| 4 | Barrier self-efficacy | - Introducing the concept of barriers and lapses - An example of how to approach and overcome a barrier to physical activity (e.g., lack of time) - Describing strategies to counter potential lapses (e.g., setting reminders, creating positive cues, coping planning) | - A step-by-step approach to overcoming barriers to physical activity (1:00) | - Reflecting on physical activity goal set in Week 3 - Identifying and proposing solutions to overcome barriers to physical activity - Identifying and developing a strategy to counter potential lapses - Setting and scheduling another S.M.A.R.T. physical activity goal |
